# Supplementary material for: Detection of OXA-23 and OXA-58 in Proteus mirabilis by immunochromatographic assays
Source: Microbiol Spectr. 2026 Feb 27;14(4):e01899-25. doi: 10.1128/spectrum.01899-25 (PMC13055382; doi:10.1128/spectrum.01899-25)
Supplement: Supplemental material — Fig. S1 and S2; Table S1. [file spectrum.01899-25-s0001.pdf]

Detection of OXA-23 and OXA-58 in *Proteus mirabilis* by immunochromatographic assays

Janina Noster-Schrader, Janko Sattler, Yvonne Stoll, Martina Spille, Andreas Rump, Stephan Göttig, Sören Gatermann, and Axel Hamprecht

Supplement

Supplementary Table 1. Isolates used in this study. Given are molecular characteristics and carb-detection-algorithm, CORIS O.K.N.V.I. RESIST-5 and CORIS RESIST ACINETO results for all isolates tested. Abbreviations: ACA: amoxicillin-clavulanic acid; SAM: ampicillin-sulbactam; TCC: ticarcillin-clavulanate; TEM: temocillin; mzCIM: modified zinc-supplemented carbapenem inactivation method.

**Isolate**	**Sequence Type**	**Carba-penemase**[1]	**Other β-lactama-ses**	**Other resistance genes**	**Results CPP detection algorithm (inhibition zone diameter [mm])**					**Result O.K.N.V.I. RESIST-5**	**Result RESIST ACINETO**
					ACA	SAM	TCC	TEM	mzCIM		
Carb-01	142	bla_{OXA-23}		*aph(3'')-Ib, aph(6)-Id, floR, sul2, aac(3)-IIa, aph(3')-Ia, tet(J), dfrA1, ant(3'')-Ia*	6	6	6	21	6		OXA-23
Carb-02	207	bla_{OXA-58}	bla_{CMY-2}	*cat, aadA14, tet(J), sul3, ant(3'')-Ia, dfrA1*	6	6	6	6	6		OXA-40 / 58
Carb-03	488	bla_{OXA-58}		*cat, aadA14, tet(J), ant(3'')-Ia*	6	6	6	6	6		OXA-40 / 58
Carb-04	207	bla_{OXA-58}	bla_{CMY-2}	*cat, aadA14, tet(J), dfrA1, ant(3'')-Ia, sul3*	6	6	6	6	22		
Carb-05	487	bla_{OXA-58}		*cat, aadA14, tet(J)*	6	6	6	6	6		OXA-40 / 58
Carb-06	446	$bla_{OXA-181}$	bla_{TEM-1B}, bla_{VEB-6}	*cat, tet(J), tet(A), aph(3')-Ia, sul2, aph(3'')-Ib, aph(6)-Id, catA1, ant(3'')-Ia, dfrA1, sul1, qacE, tet(A), sul1, qacE, dfrA1, ant(2'')-Ia, aac(6')-Ib*	6	6	6	6	6	OXA-48	
Carb-07	178	bla_{OXA-58}		*qacE, sul1, mph(A), tet(A), ant(3'')-Ia, aadA14, dfrA1, tet(J), cat*	6	6	6	6	6		OXA-40 / 58

Carb-08	132	*bla*$_{OXA-58}$		*ant(3'')-Ia, dfrA1, qnrD1*	6	6	6	6	17		OXA-40 / 58
Carb-09	485	*bla*$_{IMP-1}$	*bla*$_{TEM-1B}$, *bla*$_{CTX-M-15}$, *bla*$_{OXA-1}$, *bla*$_{OXA-1}$	*cat, tet(J), sul2, aph(3'')-Ib, aph(6)-Id, aac(3)-IIa, aac(6')-Ib-cr, mph(E), msr(E), sul1, qacE, ant(3'')-Ia, sul1, qacE, aadA2, dfrA16, sul1, qnrA1*	6	11	18	18	6	IMP	
Carb-10	207	*bla*$_{OXA-58}$		*cat, sul2, tet(J), sul3, ant(3'')-Ia, dfrA1, qnrD1*	6	6	6	6	6		OXA-40 / 58
Carb-11	479	*bla*$_{OXA-48}$	*bla*$_{CTX-M-14b}$	*cat, tet(J), aph(6)-Id, aph(3'')-Ib, ant(3'')-Ia, dfrA1, aph(3')-VIb, aph(3'')-Ib, aph(6)-Id, aph(3')-Ia, aac(3)-IIa, aph(3'')-Ib*	6	6	6	6	6	OXA-48	
Carb-12	423	*bla*$_{OXA-58}$		*cat, aadA14, tet(J), qnrD1*	6	6	6	6	6		OXA-40 / 58
Carb-13	142	*bla*$_{OXA-23}$		*sul2, floR, aph(6)-Id, aph(3'')-Ib, aph(3')-Ia, aac(3)-IIa, aph(3')-Ia, tet(J), ant(3'')-Ia, dfrA1*	6	6	6	21	6		OXA-23
Carb-14	484	*bla*$_{OXA-48}$	*bla*$_{TEM-1B}$	*cat, tet(J), sul1, qacE, ant(3'')-Ia, dfrA1, aph(6)-Id, aph(3'')-Ib, sul2, catA1*	6	10	6	6	6	OXA-48	
Carb-15	142	*bla*$_{OXA-23}$		*sul2, aph(6)-Id, aph(3'')-Ib, aph(3')-VIa, aph(3')-Ia, aac(3)-IIa, tet(J), ant(3'')-Ia, dfrA1*	6	6	6	22	6		OXA-23
Carb-16	135	*bla*$_{OXA-48}$	*bla*$_{TEM-1B}$, *bla*$_{TEM-2}$, *bla*$_{CTX-M-14b}$	*cat, tet(J), catA1, dfrA12, aadA2, qacE, sul1, mph(A), sul2, aph(3'')-Ib, aph(6)-Id, aac(3)-IId, sul1, qacE, aadA5, dfrA17, ant(3'')-Ia, dfrA1*	6	6	6	6	6	OXA-48	
Carb-17	135	*bla*$_{OXA-23}$	*bla*$_{CMY-138}$, *bla*$_{VEB-6}$, *bla*$_{TEM-1B}$, *bla*$_{TEM-2}$	*cat, tet(J), ant(3'')-Ia, dfrA1, aph(6)-Id, ant(2'')-Ia, dfrA1, qacE, sul1, tet(A), qacE, sul1, aph(3')-Ia, qacE, cmlA1, ant(2'')-Ia, aac(6')-Ib*	6	6	6	6	6		OXA-23
Carb-18	178	*bla*$_{OXA-48}$	*bla*$_{CARB-2}$, *bla*$_{OXA-10}$, *bla*$_{CTX-M-14b}$	*ant(3'')-Ia, dfrA1, cat, tet(J), ant(3'')-Ia, dfrA1, sul1, qacE, tet(B), sul1, qacE, ant(3'')-Ia, ant(2'')-Ia, aph(3')-Ia, aph(3'')-Ib, aph(3')-VIb, aph(3'')-Ib*	6	6	6	6	6	OXA-48	
Carb-19	142	*bla*$_{OXA-23}$		*sul2, floR, aph(6)-Id, aph(3'')-Ib, aph(3')-Ia, aac(3)-IIa, aph(3')-Ia, tet(J), ant(3'')-Ia, dfrA1*	6	6	6	22	6		OXA-23
Carb-20	446	*bla*$_{OXA-181}$	*bla*$_{TEM-1B}$, *bla*$_{VEB-6}$	*ant(3'')-Ia, dfrA1, cat, tet(J), tet(A), aph(6)-Id, aph(3'')-Ib, sul2, catA1, ant(3'')-Ia, dfrA1, sul1, qacE, tet(A), sul1, qacE, qnrA1, sul1, qacE, dfrA1, ant(2'')-Ia, aac(6')-Ib*	6	6	6	10	6	OXA-48	

Carb-21	142	*bla*$_{OXA-23}$		*sul2, aph(6)-Id, aph(3'')-Ib, aph(3')-Ia, tet(J), ant(3'')-Ia, dfrA1*	6	6	6	23	6			OXA-23
Carb-22	142	*bla*$_{OXA-23}$		*sul2, floR, aph(6)-Id, aph(3'')-Ib, aph(3')-Ia, aac(3)-IIa, aph(3')-Ia, tet(J), ant(3'')-Ia, dfrA1*	6	6	12	23	6			OXA-23
Carb-23	142	*bla*$_{OXA-23}$		*tet(J), aph(3')-Ia, aac(3)-IIa, aph(3')-Ia, aph(3'')-Ib, aph(6)-Id, floR, sul2, ant(3'')-Ia, dfrA1*	6	6	6	21	6			OXA-23
Carb-24	142	*bla*$_{OXA-23}$		*aph(3'')-Ib, aph(6)-Id, floR, sul2, aac(3)-IIa, aph(3')-Ia, tet(J), dfrA1, ant(3'')-Ia*	6	6	10	21	6			OXA-23
Carb-25	135	*bla*$_{OXA-48}$	*bla*$_{CTX-M-65}$, *bla*$_{TEM-1B}$, *bla*$_{OXA-1}$	*cat, tet(J), catA1, dfrA17, aadA5, qacE, sul1, fosA3, aph(3')-Ia, sul2, aph(3'')-Ib, aph(6)-Id, aac(3)-IId, sul1, qacE, ARR-3, catB3, aac(6')-Ib-cr, aac(3)-IVa, aph(4)-Ia, sul2, floR, ant(3'')-Ia, dfrA1*	6	8	6	6	24			
Carb-26	92	*bla*$_{OXA-162}$	*bla*$_{CTX-M-15}$	*tet(J)*	6	6	6	6	6		OXA-48	
Carb-27	234	*bla*$_{OXA-48}$	*bla*$_{CTX-M-14b}$, *bla*$_{TEM-1B}$	*tet(J), rmtB, dfrA17, aadA5, qacE, sul1, mph(A), aph(6)-Id, aph(3'')-Ib, sul2, aph(3')-Ia, qnrD1*	6	6	6	6	6		OXA-48	
Carb-28	142	*bla*$_{OXA-23}$		*sul2, floR, aph(6)-Id, aph(3'')-Ib, aph(3')-Ia, aac(3)-IIa, aph(3')-Ia, tet(J), ant(3'')-Ia, dfrA1*	6	6	6	19	6			OXA-23
Carb-29	482	*bla*$_{OXA-181}$		*tet(J), qnrS1*	6	6	6	6	6		OXA-48	
Carb-30	140	*bla*$_{OXA-58}$		*tet(J), sul3, ant(3'')-Ia, cmlA1, aadA2, ant(3'')-Ia, dfrA1, aadA14, qnrD1*	6	6	6	6	26			OXA-40 / 58
Carb-31	481	*bla*$_{OXA-58}$		*tet(J), aadA14*	6	6	6	6	6			OXA-40 / 58
Carb-32	343	*bla*$_{OXA-48}$		*cat, tet(J), catA3, dfrA1*	12	10	11	8	6		OXA-48	
Carb-33	93	*bla*$_{VIM-1}$	*bla*$_{SHV-12}$, *bla*$_{TEM-1B}$, *bla*$_{TEM-1B}$, *bla*$_{CMY-99}$, *bla*$_{TEM-1A}$, *bla*$_{OXA-9}$	*cat, tet(J), ant(3'')-Ia, aac(6')-Ib, aph(6)-Id, aph(3'')-Ib, sul2, ant(3'')-Ia, dfrA1, aac(6')-Il, mph(E), msr(E), armA, sul1, qacE, aadA2, dfrA12, aac(6')-Il, dfrA1, ant(3'')-Ia, sul2, aph(3'')-Ib, aph(6)-Id, catA1, ant(3'')-Ia, dfrA1*	6	6	12	20	6		VIM	
Carb-34	479	*bla*$_{OXA-48}$	*bla*$_{CTX-M-14b}$	*tet(J), cat, dfrA1, ant(3'')-Ia, aac(3)-IIa, aph(3')-Ia, aph(6)-Id, aph(3'')-Ib, aph(3')-VIb, aph(3'')-Ib, aph(3'')-Ib, aph(6)-Id*	6	6	6	6	6		OXA-48	
Carb-35	461	*bla*$_{OXA-48}$		*tet(J), dfrA1, cat*	6	6	12	6	6		OXA-48	

Carb-36	477	*bla*$_{NDM-1}$	*bla*$_{TEM-1B}$	*cat, tet(J), aph(6)-Id, aph(3'')-Ib, sul2, sul1, qacE, dfrA7, catA1*	13	6	11	22	6	NDM	NDM
Carb-37	477	*bla*$_{NDM-1}$	*bla*$_{TEM-1B}$	*aph(6)-Id, aph(3'')-Ib, sul2, sul1, qacE, dfrA7, catA1, cat, tet(J)*	6	6	6	24	6	NDM	NDM
Carb-38	135	*bla*$_{VIM-1}$, *bla*$_{VIM-4}$	*bla*$_{TEM-2}$	*cat, tet(J), sul2, qacE, sul1, qnrA1, ant(3'')-Ia, aph(3')-VI, catA1, aph(3')-Ia, aac(3)-IVa, lnu(F), aac(6')-IIc, dfrA1*	6	6	18	19	6	VIM	
Carb-39	322	*bla*$_{OXA-58}$		*sul2, aadA14, cat, tet(J)*	6	6	6	6	6		OXA-40 / 58
Carb-40	426	*bla*$_{OXA-58}$		*aadA14, ant(3'')-Ia, tet(J), cat*	6	6	6	28	6		OXA-40 / 58
Carb-41	312	*bla*$_{KPC-3}$		*dfrA1, catA1, sul1, qacE, cat, tet(J)*	6	6	6	28	12	KPC	
Carb-42	142	*bla*$_{OXA-23}$		*aph(6)-Id, aph(3'')-Ib, aac(3)-IIa, aph(3')-Ia, tet(J), ant(3'')-Ia, dfrA1*	13	6	11	23	6		OXA-23
Carb-43	142	*bla*$_{OXA-23}$		*aph(3')-VIa, aph(3'')-Ib, aph(6)-Id, sul2, aac(3)-IIa, aph(3')-Ia, tet(J), ant(3'')-Ia, dfrA1*	6	6	6	20	6		OXA-23
Carb-44	142	*bla*$_{OXA-23}$		*sul2, aph(6)-Id, aph(3'')-Ib, aph(3')-VIa, aac(3)-IIa, aph(3')-Ia, tet(J), ant(3'')-Ia, dfrA1*	6	6	6	19	6		OXA-23
Carb-46	199	*bla*$_{NDM-1}$	*bla*$_{OXA-1}$	*floR, aph(6)-Id, aph(3'')-Ib, sul2, ARR-3, cat, tet(C), qnrD1, dfrA32, ere(A), aac(6')-Ib-cr, aph(3')-Ia, dfrA12, aadA2, qacE, tet(J)*	6	6	17	19	6	NDM	NDM
Carb-47	132	*bla*$_{NDM-1}$	*bla*$_{OXA-10}$, *bla*$_{CMY-16}$	*floR, tet(A), aph(6)-Id, aph(3'')-Ib, sul2, qnrD1, cmlA1, aph(3')-Ia, ARR-3, dfrA14, sul1, aph(3')-VIa, aadA1, qacE, dfrA1*	6	6	14	21	6	NDM	NDM
Carb-48	142	*bla*$_{OXA-23}$		*sul2, floR, aph(6)-Id, aph(3'')-Ib, tet(J), ant(3'')-Ia, dfrA1*	6	6	6	18	6		OXA-23
Carb-49	178	*bla*$_{OXA-48}$	*bla*$_{OXA-10}$, *bla*$_{CARB-2}$	*ant(2'')-Ia, dfrA1, qacE, sul1, ant(3'')-Ia, tet(J)*	6	6	6	6	6	OXA-48	
Carb-50	135	*bla*$_{VIM-78}$	*bla*$_{CTX-M-15}$, *bla*$_{TEM-1A}$	*cat, catA1, mph(E), msr(E), armA, sul2, ant(3'')-Ia, aadA5, dfrA17, sul1, qacE, lnu(F), dfrA1, aph(3')-Ia, tet(J)*	6	6	18	14	6	VIM	
Carb-51	142	*bla*$_{OXA-23}$		*sul2, aph(6)-Id, aph(3'')-Ib, aph(3')-Ia, tet(J), dfrA1, ant(3'')-Ia*	6	6	6	25	6		OXA-23
Carb-52	142	*bla*$_{OXA-23}$	*bla*$_{CMY-138}$, *bla*$_{TEM-2}$	*mph(E), ant(3'')-Ia, dfrA1, aph(3')-Ia, tet(J), aph(3'')-Ib, aph(6)-Id, sul2, ant(2'')-Ia, cmlA1, qacE, sul1*	6	6	14	25	6		OXA-23
Carb-53	207	*bla*$_{OXA-58}$		*cat, sul2, aadA14, dfrA1, ant(3'')-Ia, sul3, tet(J)*	6	6	6	6	6		OXA-40 / 58

Carb-54	142	*bla*OXA-23		*sul2, aph(6)-Id, aph(3'')-Ib, aph(3')-VIa, aac(3)-IIa, tet(J), aph(3')-Ia, ant(3'')-Ia, dfrA1*	6	6	6	26	6		OXA-23
Carb-55	142	*bla*OXA-23		*sul2, aph(6)-Id, aph(3'')-Ib, aph(3')-VIa, dfrA1, ant(3'')-Ia, aac(3)-IIa, aph(3')-Ia, tet(J)*	6	6	6	22	6		OXA-23
Carb-56	135	*bla*VIM-78	*bla*CTX-M-15, *bla*TEM-1A	*cat, catA1, mph(E), msr(E), armA, sul1, qacE, aph(3')-Ia, ant(2'')-Ia, dfrA1, ant(3'')-Ia, tet(J)*	6	6	8	21	6	VIM	OXA-23
Carb-57	479	*bla*OXA-48	*bla*TEM-1B	*tet(J), catA1, aac(3)-IIa, sul2, aph(6)-Id, aph(3')-Ia, ant(3'')-Ia, dfrA1, cat*	6	6	9	26	6	OXA-48	
Carb-58	192*	*bla*NDM-5	*bla*TEM-1B	*catA1, dfrA7, qacE, sul1, sul2, aph(3'')-Ib, aph(6)-Id, qnrD1, tet(J)*	6	14	24	18	6	NDM	NDM
Carb-59	142	*bla*OXA-23		*aph(3')-Ia, sul2, aph(6)-Id, aph(3'')-Ib, aph(3')-VIa, aac(3)-IIa, tet(J), ant(3'')-Ia, dfrA1*	6	6	10	18	6		OXA-23
Carb-60	178	*bla*OXA-181		*qnrS1, dfrA1, ant(3'')-Ia, tet(J), dfrA15, qacE, sul1, cat*	8	6	6	14	6	OXA-48	
Carb-61	135	*bla*VIM-78	*bla*CTX-M-15	*tet(J), cat, qacE, sul1, armA, msr(E), mph(E), catA1, aph(3')-Ia, dfrA1, ant(3'')-Ia*	12	6	12	22	6	VIM	
Carb-62	135	*bla*VIM-4, , *bla*VIM-75	*bla*CTX-M-15, *bla*TEM-2	*cat, catA1, mph(E), msr(E), armA, sul1, qacE, aph(3')-VI, aac(6')-IIc, aph(3'')-Ib, aph(6)-Id, aac(3)-IId, aph(3')-Ia, ant(3'')-Ia, dfrA1, tet(J)*	8	6	8	22	6	VIM	
Carb-63	269	*bla*VIM-1, *bla*OXA-48	*bla*CTX-M-14b, *bla*TEM-2	*cat, aph(6)-Id, tet(J), catA1, sul2, aac(6')-II, aph(3')-VIb, aph(3')-Ia, aph(3'')-Ib, dfrA1*	6	6	6	6	6	OXA-48, VIM	
Carb-64	178	*bla*OXA-48	*bla*OXA-10, *bla*CARB-2	*tet(B), dfrA1, ant(2'')-Ia, sul1, qacE, tet(J), cat*	6	6	6	13	6	OXA-48	
Carb-65	185	*bla*KPC-3	*bla*TEM-1A, *bla*OXA-9	*aac(6')-Ib, mph(A), sul2, aph(3'')-Ib, aph(6)-Id, ant(3'')-Ia, cat, tet(J), dfrA1*	6	6	6	6	6	KPC	
Carb-66	479	*bla*OXA-48	*bla*TEM-1B	*cat, catA1, aac(3)-IIa, sul2, aph(6)-Id, aph(3')-Ia, dfrA1, ant(3'')-Ia, tet(J)*	6	6	6	20	6	OXA-48	
Carb-67	92	*bla*KPC-3		*aac(6')-Ib-cr, ARR-3, dfrA27, aadA16, qacE, sul1, qnrB6, dfrA1, aph(3')-Ia, tet(J)*	17	2	16	28	6	KPC	

Carb-68	142*	bla_{OXA-23}, bla_{KPC-3}		*aph(3')-Ia, aac(6')-Ib-cr, ARR-3, dfrA27, aadA16, qacE, sul1, qnrB6, aph(3'')-Ib, aph(6)-Id, sul2, ant(3'')-Ia, dfrA1, tet(J), aac(3)-IIa*	12	6	16	29	6	KPC	OXA-23
Carb-69	269	bla_{NDM-1}	$bla_{CTX-M-55}$, bla_{TEM-1B}	*cat, tet(J), mph(E), msr(E), armA, sul1, dfrA1, ant(3'')-Ia, aac(3)-IIa, aph(6)-Id, aph(3'')-Ib, sul2, qnrD1, catA1, aph(3')-Ia*	13	10	12	25	6	NDM	NDM
Carb-70	446	bla_{NDM-1}		*cat, ant(3'')-Ia, tet(J), mph(E), msr(E), armA, tet(A), catA1, aph(3')-Ia, sul1, dfrA1, ant(2'')-Ia, aac(6')-Ib*	13	10	12	25	6	NDM	NDM
Carb-71	134*	bla_{OXA-48}		*cat, tet(J)*	6	6	6	6	6	OXA-48	
Carb-72	269	bla_{NDM-1}	$bla_{CTX-M-55}$, bla_{TEM-1B}	*cat, tet(J), ant(3'')-Ia, dfrA1, sul1, armA, msr(E), mph(E), aac(3)-IIa, aph(6)-Id, aph(3'')-Ib, sul2, qnrD1, catA1*	8	6	14	24	6	NDM	NDM
Carb-73	269	bla_{NDM-1}	$bla_{CTX-M-55}$, bla_{TEM-1B}	*cat, sul1, armA, msr(E), mph(E), tet(J), aac(3)-IIa, aph(6)-Id, aph(3'')-Ib, sul2, qnrD1, catA1, aph(3')-Ia, dfrA1, ant(3'')-Ia*	6	6	16	24	6	NDM	NDM
Carb-74	222	bla_{NDM-1}	bla_{TEM-2}, bla_{OXA-10}, bla_{CMY-16}	*floR, tet(A), aph(6)-Id, aph(3'')-Ib, sul2, cat, qnrA6, dfrA14, ARR-3, cmlA1, ant(3'')-Ia, qacE, tet(J), aph(3')-VI*	6	6	16	26	6	NDM	NDM
Carb-75	269	bla_{VIM-1}	bla_{TEM-2}	*floR, tet(A), aph(6)-Id, aph(3'')-Ib, sul2, cat, qnrA6, dfrA14, ARR-3, cmlA1, ant(3'')-Ia, qacE, tet(J), aph(3')-VI*	10	6	18	28	6	VIM	
Carb-76	178	bla_{OXA-48}	bla_{CARB-2}, bla_{OXA-10}	*ant(2'')-Ia, qacE, sul1, dfrA1, cat, tet(J)*	6	6	12	20	6	OXA-48	
Carb-77	269	bla_{NDM-1}	$bla_{CTX-M-55}$, bla_{TEM-1B}	*cat, mph(E), msr(E), armA, sul1, ant(3'')-Ia, dfrA1, aac(3)-IIa, aph(6)-Id, aph(3'')-Ib, sul2, qnrD1, catA1, aph(3')-Ia, tet(J)*	8	6	18	26	6	NDM	NDM
Carb-78	135	bla_{VIM-78}	$bla_{CTX-M-55}$, bla_{TEM-1A}	*cat, catA1, armA, msr(E), mph(E), sul2, ant(3'')-Ia, aadA5, dfrA17, aph(3')-Ia, qacE, sul1, lnu(F), dfrA1, tet(J)*	13	6	18	18	6	VIM	
Carb-79	348	$bla_{OXA-181}$		*cat, qnrS1, tet(J)*	6	6	6	6	6	OXA-48	
Carb-80	269	bla_{NDM-1}	$bla_{CTX-M-55}$, bla_{TEM-1B}	*cat, dfrA1, ant(3'')-Ia, tet(J), sul1, armA, msr(E), mph(E), aac(3)-IIa, aph(6)-Id, aph(3'')-Ib, sul2, qnrD1, catA1, aph(3')-Ia*	9	6	13	26	6	NDM	NDM
Carb-81	142	bla_{OXA-23}		*aph(3'')-Ib, aph(6)-Id, floR, sul2, tet(J), dfrA1, ant(3'')-Ia*	6	6	10	25	6		OXA-23

Carb-82	446	*bla*$_{OXA-48}$	*bla*$_{VEB-6}$	*ant(2'')-Ia, aac(6')-Ib, cat, tet(J), ant(3'')-Ia, catA1, qnrA1, qacE, sul1, dfrA1*	6	6	6	6	6	OXA-48	
Carb-83	142	*bla*$_{OXA-23}$		*sul2, floR, aph(6)-Id, aph(3'')-Ib, ant(3'')-Ia, dfrA1, aac(3)-IIa, aph(3')-Ia, tet(J)*	6	6	17	26	6		OXA-23
Carb-84	446	*bla*$_{OXA-48}$	*bla*$_{VEB-6}$	*ant(2'')-Ia, aac(6')-Ib, cat, tet(J), catA1, ant(3'')-Ia, tet(A), sul1, qacE, dfrA1*	6	6	6	6	6	OXA-48	
Carb-85	269	*bla*$_{NDM-1}$	*bla*$_{CTX-M-55}$, *bla*$_{TEM-1B}$	*cat, ant(3'')-Ia, dfrA1, tet(J), mph(E), msr(E), armA, sul1, aac(3)-IIa, aph(6)-Id, aph(3'')-Ib, sul2, catA1, qnrD1, aph(3')-Ia*	12	6	10	24	6	NDM	
Carb-86	142	*bla*$_{OXA-23}$		*sul2, floR, aph(6)-Id, aph(3'')-Ib, aac(3)-IIa, aph(3')-Ia, tet(J), ant(3'')-Ia, dfrA1*	6	6	6	6	6		OXA-23
Carb-87	135	*bla*$_{NDM-1}$	*bla*$_{CTX-M-15}$, *bla*$_{TEM-1A}$, *bla*$_{OXA-9}$	*dfrA1, tet(J), catA1, dfrA17, aadA5, qacE, sul1, armA, msr(E), mph(E), sul2, aac(6')-Ib, qnrS1, aph(3')-VI, ant(3'')-Ia, cat*	11	6	19	24	6		NDM
Carb-88	222	*bla*$_{NDM-1}$	*bla*$_{TEM-2}$, *bla*$_{OXA-10}$, *bla*$_{CMY-16}$	*floR, tet(A), aph(6)-Id, aph(3'')-Ib, sul2, cat, aph(3')-VIa, qacE, ant(3'')-Ia, cmlA1, ARR-3, dfrA14, qnrA6, tet(J)*	6	6	14	24	6	NDM	NDM
Carb-89	135	*bla*$_{NDM-1}$, *bla*$_{OXA-48}$	*bla*$_{CTX-M-15}$, *bla*$_{TEM-1A}$, *bla*$_{VEB-6}$, *bla*$_{OXA-9}$	*tet(A), qnrA1, tet(J), dfrA1, ant(3'')-Ia, aac(6')-Ib, catA1, dfrA17, aadA5, qacE, sul1, armA, msr(E), mph(E), aph(3')-VI, qnrS1, sul2, cat*	6	6	12	10	6	NDM, OXA-48	
Carb-90	269	*bla*$_{NDM-1}$	*bla*$_{CTX-M-55}$, *bla*$_{TEM-1B}$	*cat, mph(E), msr(E), armA, sul1, aac(3)-IIa, sul2, aph(3'')-Ib, aph(6)-Id, tet(J), catA1, qnrD1, ant(3'')-Ia, dfrA1, aph(3')-Ia*	6	6	8	24	6	NDM	NDM
Carb-91	759*	*bla*$_{NDM-1}$	*bla*$_{OXA-1}$	*tet(J), cat, sul2, aph(3'')-Ib, aph(6)-Id, msr(E), mph(E), mph(A), qnrD3, tet(C), dfrA32, ere(A), aadA2, aac(6')-Ib-cr, catB3, ARR-3, catA2, aph(3')-Ia, ant(2'')-Ia*	6	6	26	29	6	NDM	NDM

* imperfect sequence type matches

[1]according to DNA sequencing results

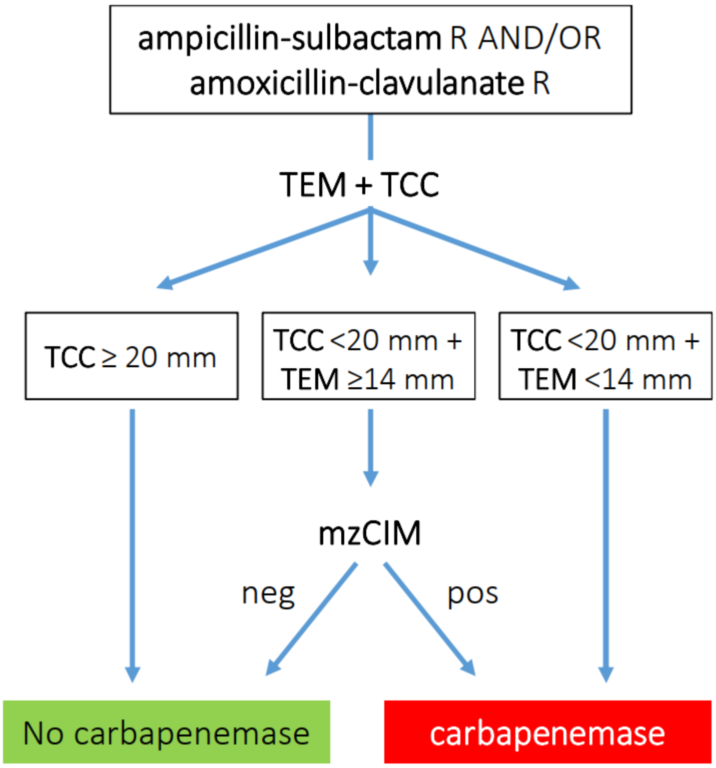

Supplementary Figure 1. Algorithm for detection of carbapenemases in P. mirabilis, reprinted in unmodified form from Hamprecht et al., Clinical Microbiology and Infection Volume 29, Issue 9, September 2023, Pages 1198.e1-1198.e6 (CC BY-NC-ND 4.0, https://creativecommons.org/licenses/by-nc-nd/4.0/)

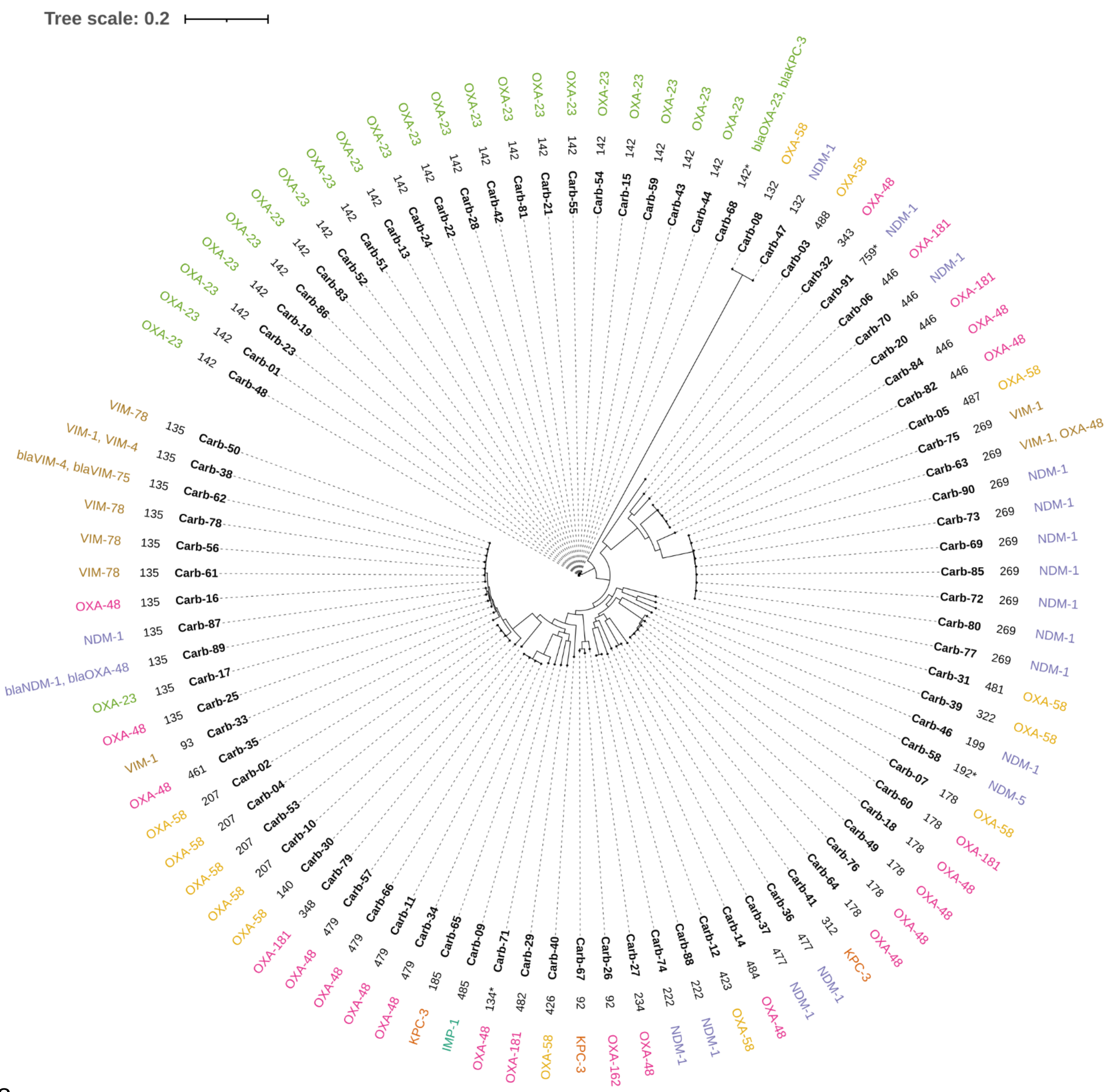

Supplementary Figure 2. Phylogenetic relatedness of all 90 isolates based on whole genome SNP phylogeny, including carbapenemase type (outer circle) and sequence type (middle circle).
